# St. Bartholomew's Hospital

**Published:** 1906-03-31

**Authors:** 


### ST. BARTHOLOMEW'S HOSPITAL.

The acting editor of "Burdett's Hospitals and Charities, 1906," desires us to correct, with an expression of his regret, a printer's error on page 300, where the name of Lord Ludlow has been wrongly inserted, and the name of the new clerk, Mr. Thomas Hoyes, has been omitted altogether.

